# From checkpoint to checkpoint: DNA damage ATR/Chk1 checkpoint signalling elicits PD-L1 immune checkpoint activation

**DOI:** 10.1038/s41416-018-0017-x

**Published:** 2018-03-13

**Authors:** Kent W. Mouw, Panagiotis A. Konstantinopoulos

**Affiliations:** 1 Department of Radiation Oncology, Dana-Farber Cancer Institute/Brigham & Women’s Hospital, Harvard Medical School, Boston, MA 02215 USA; 2 Gynecologic Medical Oncology Program, Dana-Farber Cancer Institute, Harvard Medical School, Boston, MA 02215 USA

**Keywords:** Cancer, Oncology

## Abstract

Multiple clinical studies have revealed a link between genomic instability and response to anti-PD-1/PD-L1 therapy in cancer management. A recent study has revealed an important role for the ATR/Chk1 DNA damage checkpoint in regulating PD-L1 expression, raising important clinical and translational questions for therapy selection and study design.

Genomic instability, driven by exogenous damaging agents or inherent DNA repair defects, has numerous effects on cell signalling and behaviour, and DNA damage-induced mutations directly contribute to tumourigenesis and drive the tumour phenotype. The association between genomic instability and the immune system has recently come under intense scrutiny given the discovery and clinical implementation of drugs that target the immune checkpoint response.^1^ Although it is evident that tumour DNA damage and repair processes can impact the host immune landscape and drive sensitivity to immune checkpoint inhibitors, the cellular mechanisms underlying this association are poorly understood. A recent report by Sato et al. begins to shed light on the molecular underpinnings of signal transduction from DNA damage to the immune checkpoint, and identifies a key role for the DNA damage sensor kinases ATM, ATR, and Chk1 in this process (Fig. 1).^2^

**Fig. 1:**
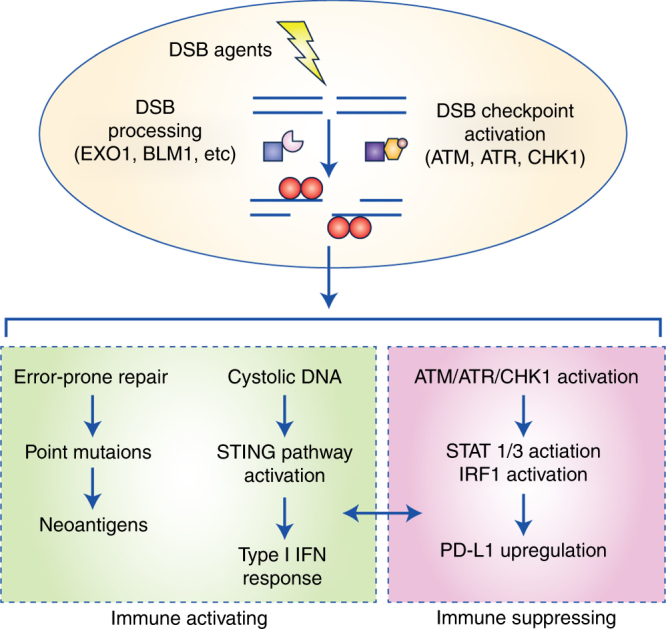
Immune activating and suppressive effects of DNA double-strand break (DSB) signalling. DSBs created by damaging agents such as ionizing radiation activate a network of signalling pathways that balance the host immune response. If repaired using an error-prone pathway such as non-homologous end joining (NHEJ) or alternative end joining, DSBs can result in somatic mutations that act as neoantigens. DNA damage can also activate the innate immune system via the STING pathway.^15^ DSB-mediated immune activation is balanced by concomitant inhibitory signalling, including upregulation of PD-L1 expression. Sato et al. show that DNA damage signalling via the checkpoint kinases ATM, ATR, and Chk1 drive PD-L1 expression on tumour cells via STAT1/STAT3/IRF3 activation

Multiple lines of clinical evidence have revealed a link between genomic instability and response to anti-PD1/PD-L1 therapy. For example, an association between increased tumour mutational burden and improved response to pembrolizumab was identified in a cohort of non-small cell lung cancer patients^3^. Importantly, several of the tumours with the highest mutational burden harboured a deleterious mutation in one or more DNA repair genes, such as *BRCA2* or *MSH2*. These observations supported the idea that loss of DNA repair fidelity can drive mutagenesis, the creation of tumour-specific neoantigens, and the activation of a host anti-tumour immune response.

The association between repair deficiency and improved response to PD-1/PD-L1 blockade has now been demonstrated for several DNA repair pathways. Tumours with mismatch repair (MMR) pathway deficiency exhibit a microsatellite instability (MSI) phenotype, and are characterised by a high mutation burden, an activated immune microenvironment, and increased PD-1/PD-L1 expression on tumour and immune/stromal cells. As such, MSI tumours exhibit high response rates to anti-PD1/PD-L1 therapy,^4^ and pembrolizumab (anti-PD1) was recently approved by the FDA for treatment of patients with advanced MMR-deficient tumours.

Associations between alterations in other DNA repair pathways and tumour immune features have also been reported. Tumours with modifications in the homologous recombination (HR) pathway (including *BRCA1, BRCA2*, and *PALB2*) have increased mutation burden, predicted neoantigen load, and PD-1/PD-L1 expression. This raises the possibility that HR-deficient tumours may be sensitive to PD-1/PD-L1 inhibitors; however, their response to these agents appears less robust compared with MMR-deficient or other hypermutated cancers, such as melanomas and non-small cell lung cancers.^5^ In addition, tumours with mutations in the exonuclease (proofreading) domain of polymerase epsilon (POLE) or polymerase delta (POLD1) have among the highest mutation burdens identified to date, and *POLE/POLD1*-mutated tumours exhibit high levels of immune activation and PD-1/PD-L1 expression, and are sensitive to immune checkpoint blockade.^6, 7^

Despite these robust clinical associations, the mechanisms linking DNA damage to increased PD-L1 expression and immunotherapy response are incompletely understood. To functionally characterise the impact of DNA damage on PD-L1 expression, Sato et al. subjected several DNA repair-proficient cancer cell lines to ionizing radiation, double strand break (DSB)-inducing drugs such as etoposide, or a poly(ADP)-ribose polymerase inhibitor (PARPi). Treatment resulted in a time-dependent increase in PD-L1 expression at both the transcriptional and protein levels. To investigate the role of the DNA damage response in this process, they added specific inhibitors of ATM, ATR, or Chk1, and demonstrated that inhibition of any of these DNA damage checkpoint kinases could suppress PD-L1 upregulation. Next, the authors screened an siRNA library targeting DNA repair genes; they found that depletion of *BRCA2*, *PALB2* and *XRCC5* [encoding Ku80, involved in non-homologous end joining (NHEJ)] resulted in the largest increase in radiation-induced PD-L1 expression. Again, they observed that pharmacologic inhibition of ATM, ATR, or Chk1 was sufficient to abrogate PD-L1 upregulation. Interestingly, PD-L1 upregulation in Ku80-depleted cells could also be abrogated by depletion of either *EXO1* (exonuclease) or *BLM* (helicase); two genes that are required for DSB processing and end resection. Similarly, in their screen, loss of *BRCA1* did not show a significant increase in ionizing radiation-induced PD-L1 expression, compared with control cells. This highlights the importance of Chk1 activation via end resection as a key process in DSB-induced PD-L1 expression. Although both *BRCA1* and *BRCA2* are required for HR, they have distinct roles in this process; *BRCA1* promotes DNA end resection by relieving the barrier posed by 53BP1 in HR, a process where *BRCA2* is not involved. Therefore, according to their model, DNA end resection is impaired in *BRCA1*-defective cells, meaning ATR/Chk1 signalling is not effectively activated and PD-L1 activation does not occur. This is in stark contrast to *BRCA2*-defective cells where DNA end resection is not impaired as *BRCA2* is not involved in this process. Finally, the authors showed that damage-induced ATM/ATR/Chk1-mediated PD-L1 upregulation is dependent on IRF1 signalling through phosphorylated STAT1/3.

The work by Sato et al. provides important mechanistic insights linking DNA damage with immune activation, by identifying a critical role for the DNA damage checkpoint in regulating PD-L1 expression. Although these data have provided a window into one aspect of the signalling network that links genomic instability with immune signalling, the molecular details of much of the network remain to be elucidated. Particularly, the events downstream of ATR activation, including transcriptional changes and/or direct activation (or repression) of other signalling factors, may uncover additional mechanisms through which the immune response can be modulated by DNA damage.

Temporal aspects of DNA damage (or repair deficiency) may also impact the immune response: in the experiments by Sato et al., changes in PD-L1 levels were measured following acute damage exposure or siRNA-mediated depletion of a repair factor, and the observed PD-L1 increase did not persist beyond 14 days. Therefore, the dynamics of PD-L1 expression following chronic damage exposure or in a constitutively DNA repair-deficient background (such as *BRCA2*^-/-^ cells) are unclear. In addition, many of these experiments will ultimately have to be conducted in immune-competent model systems in order to fully understand the breadth of the immune response to DNA damage.

This work also has several important clinical implications. The optimal timing and combinations of DNA damaging agents with immunotherapy are not known. Furthermore, both immune stimulating and suppressive effects of combined regimens have been noted.^8^ Numerous combinations of DSB-inducing agents with immune checkpoint inhibitors have entered clinical trial evaluation in various disease settings, including combinations of immune checkpoint inhibitors with targeted agents (such as PARPi), radiation therapy, or DSB-inducing chemotherapy agents.^1^ The data by Sato et al. raise the possibility that, at least in the setting of exposure to DSB-inducing agents, DNA damage checkpoint inhibitors (targeting ATR, ATM, or Chk1) may decrease tumour response to immune checkpoint inhibition by suppressing PD-L1 expression, thereby arguing against a triplet of DSB-inducing therapy/ATR-Chk1 blockade/PD-1-PD-L1 blockade. Nonetheless, the optimal sequence of conventional or targeted agents with anti-PD1/PD-L1 therapy may be highly context-dependent, and this work highlights the important role for pre-clinical studies in identifying potential mechanisms of synergy or antagonism.

Another clinical implication of this work is the selection of anticancer therapy after PARPi progression. PARPi are synthetically lethal with HR deficiency and are now FDA approved for clinical use in ovarian cancer, while also being evaluated in several other HR-deficient tumour settings. However, a substantial fraction of patients eventually develop resistance to PARP inhibition and several mechanisms of PARPi resistance are now emerging. In *BRCA1*-mutated tumours, resistance to PARPi may occur due to a rescue of DNA end resection ability via loss of 53BP1, REV7, or Ku70/80, which increase HR capacity.^9–11^ In this setting, re-establishing end resection may promote PD-L1 upregulation via EXO1/BLM (and ATM/ ATR/ Chk1) activity, and thus sensitise tumours to PD-1/PD-L1 blockade with or without DSB-inducing agents. In *BRCA2*-mutated tumours, resistance to PARPi may occur via protection of the replication fork,^12, 13^ which is dependent on ATR activity.^14^ This ATR activity may lead to upregulation of PD-L1 and promote response to PD-1/PD-L1 blockade. In both scenarios of PARPi resistance in BRCA1/2-mutated tumours, the study by Sato et al. suggests that PD-1/PD-L1 inhibitors may be a useful therapeutic strategy (with or without concurrent DNA damaging agents) for tumours that have progressed on PARPi.

The work by Sato et al. highlights an important role for the DNA damage ATR/Chk1 checkpoint in regulating PD-L1 expression, thus linking DNA DSB signalling with regulation of the immune response. These observations have important clinical implications for therapy selection, particularly following progression on PARPi and other DNA damaging agents. They also have translational implications for the design of appropriate correlative studies in ongoing and future clinical trials of DNA damaging agents in combination with immunotherapy.
